# Supplementary material for: One Single Nucleotide Polymorphism of the TRPM2 Channel Gene Identified as a Risk Factor in Bipolar Disorder Associates With Autism Spectrum Disorder in a Japanese Population
Source: Diseases. 2020 Feb 7;8(1):4. doi: 10.3390/diseases8010004 (PMC7151227; doi:10.3390/diseases8010004)
Supplement: Supplementary file 1 [file diseases-08-00004-s001.pdf]

Supplementary Table 1

Cases								
**SNPs**	**Genotype /Allele**	**Real #**	**Frequency**	**Apply Hardy-Weinberg**		**Chi-square value**	**n**	***p***
				Individuals	Frequency			
rs 933151	AG	88	60.68965517	84.972414	0.5860166	1.94	145	0.16367
	AG	46	31.72413793	52.055172	0.3590012			
	GG	11	7.586206897	7.9972414	0.0549822			
	A	222	76.55172414				290	
	G	68	23.44827586					

Chi square goodness-of-fit tests with continuity correction, X_c^2
Conditional chi square goodness-of-fit test, X^{*2}
Elston-Forthofer average test, A
c =0.5

rs 933151	E11	84.97241		E11*	84.88235294
	E12	52.05517		E12*	52.23529412
	E22	7.972414		E22*	7.882352941
	$X_{0.5}^2$	208.32		X^{*2}	216.9963579
		**A**	**212.6582**		

Controls								
**SNPs**	**Genotype /Allele**	**Real #**	**Frequency**	**Apply Hardy-Weinberg**		**Chi-square value**	**n**	***p***
				Individuals	Frequency			
rs 933151	AG	89	60.9589041	92.9606165	0.63671655	4.133	146	0.0420547
	AG	55	37.6712329	47.0787671	0.32245731			
	GG	2	1.36986301	5.96061644	0.04082614			
	A	233	79.7945205				292	
	G	59	20.2054795					

Chi square goodness-of-fit tests with continuity correction, X_c^2
Conditional chi square goodness-of-fit test, X^{*2}
Elston-Forthofer average test, A
c =0.5

E11	92.96061644		E11*	92.87972509	
E12	47.07876712		E12*	47.24054983	
E22	5.960616438		E22*	5.879725086	
$X_{0.5}^2$	437.613521		X^{*2}	453.8468462	
	**A**	**445.73018**			
